# The V6-V1 interpeak interval: a novel criterion for the diagnosis of left bundle branch capture

**DOI:** 10.1093/europace/euab164

**Published:** 2021-07-12

**Authors:** Marek Jastrzębski, Haran Burri, Grzegorz Kiełbasa, Karol Curila, Paweł Moskal, Agnieszka Bednarek, Marek Rajzer, Pugazhendhi Vijayaraman

**Affiliations:** 1 First Department of Cardiology, Interventional Electrocardiology and Hypertension, Jagiellonian University, Medical College, Kraków, Poland; 2 Cardiac Pacing Unit, Cardiology Department, University Hospital of Geneva, Geneva, Switzerland; 3 Department of Cardiology, Third Faculty of Medicine, Charles University and University Hospital Kralovske Vinohrady, Prague, Czech Republic; 4 Geisinger Heart Institute, Geisinger Commonwealth School of Medicine, Wilkes-Barre, PA, USA

**Keywords:** Left bundle branch pacing, Left bundle branch capture, Left ventricular septal capture, Conduction system pacing, Electrocardiogram

## Abstract

**Aims:**

We hypothesized that during left bundle branch (LBB) area pacing, the various possible combinations of direct capture/non-capture of the septal myocardium and the LBB result in distinct patterns of right and left ventricular activation. This could translate into different combinations of R-wave peak time (RWPT) in V_1_ and V_6_. Consequently, the V_6_-V_1_ interpeak interval could differentiate the three types of LBB area capture: non-selective (ns-)LBB, selective (s-)LBB, and left ventricular septal (LVS).

**Methods and results:**

Patients with unquestionable evidence of LBB capture were included. The V_6_-V_1_ interpeak interval, V_6_RWPT, and V_1_RWPT were compared between different types of LBB area capture. A total of 468 patients from two centres were screened, with 124 patients (239 electrocardiograms) included in the analysis. Loss of LVS capture resulted in an increase in V_1_RWPT by ≥15 ms but did not impact V_6_RWPT. Loss of LBB capture resulted in an increase in V_6_RWPT by ≥15 ms but only minimally influenced V_1_RWPT. Consequently, the V_6_-V_1_ interval was longest during s-LBB capture (62.3 ± 21.4 ms), intermediate during ns-LBB capture (41.3 ± 14.0 ms), and shortest during LVS capture (26.5 ± 8.6 ms). The optimal value of the V_6_-V_1_ interval value for the differentiation between ns-LBB and LVS capture was 33 ms (area under the receiver operating characteristic curve of 84.7%). A specificity of 100% for the diagnosis of LBB capture was obtained with a cut-off value of >44 ms.

**Conclusion:**

The V_6_-V_1_ interpeak interval is a promising novel criterion for the diagnosis of LBB area capture.

**What’s new?**

- The V_6_-V_1_ interpeak interval >33 ms is a novel criterion that differentiates non-selective left bundle branch (ns-LBB) capture from left ventricular septal (LVS) myocardial capture; it is accurate, easy to measure and reproducible.
- The novel concept to assess the R-wave peak time (RWPT) in V_6_ using an intra-QRS reference time point (that is R-wave peak in V_1_ rather than the pacing stimulus) circumvents the pacing latency or intraventricular conduction delay related to V_6_RWPT prolongation.
- Loss of LVS myocardial capture results in an increase in V_1_RWPT by ≥15 ms but does not impact V_6_RWPT. This indicates that the predominant pathway of right ventricular activation during ns-LBB pacing is direct transseptal route rather than retrograde right bundle branch activation.
- Loss of LBB capture results in an increase in V_6_RWPT by ≥15 ms with a specificity of 100%, and results in minimal change in V_1_RWPT.

## Introduction

Electrocardiographic diagnosis of conduction system capture remains one of the challenges of modern physiologic pacing.^1^ This is because of the phenomenon of non-selective (ns) capture, that is simultaneous activation of the conduction system and the adjacent myocardium. While sensitive and specific electrocardiogram (ECG) criteria for His-bundle pacing were recently developed and validated, similar criteria for left bundle branch (LBB) pacing are lacking.^2^ Three main types of capture are observed during LBB area pacing: non-selective (ns)-LBB, selective (s-)LBB, and left ventricular septal (LVS) myocardial capture.^3^ The QRS and V_6_ R-wave peak time (RWPT) fixed cut-off criteria proposed arbitrarily for differentiation between ns-LBB and LVS capture are widely used.^4–6^ We recently demonstrated that a V_6_ RWPT <75 ms is nearly 100% specific for LBB capture in patients with a narrow QRS or right bundle branch block (RBBB), as is V6 RWPT ≤80 ms in case of left bundle branch block (LBBB)/intra-ventricular conduction delay/escape rhythm/asystole, but that there is a great deal of overlap in V_6_ RWPT between ns-LBB and septal myocardial capture in all instances (with LBB capture possible even with values of >100ms).^7^ These criteria are likely influenced not only by the LBB capture/non-capture but also by the degree of the initial latency and the velocity of intraventricular conduction. Furthermore, no QRS-based criteria for differentiation between ns-LBB and s-LBB capture exist. Therefore, a search for new ECG markers for the differentiation of the three main types of LBB area capture is justified.

**Figure 1:**
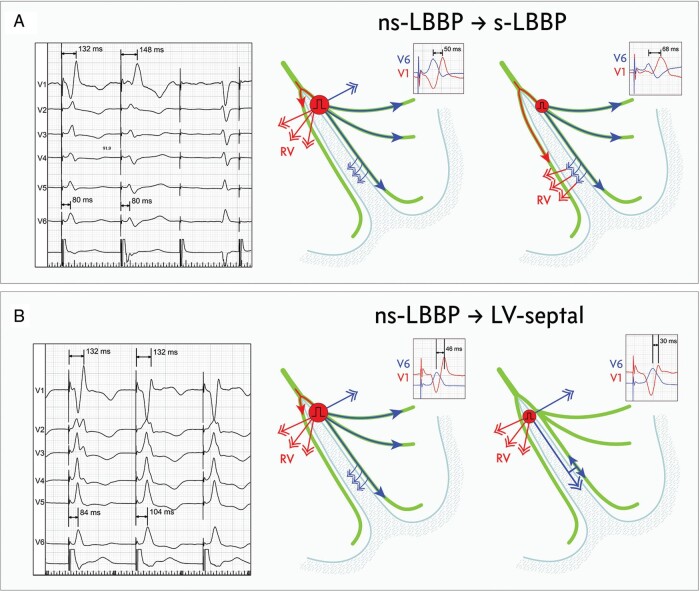
Left bundle branch (LBB) area pacing with hypothesized ventricular activation patterns during QRS morphology transitions. (*A*) Transition from non-selective LBB capture to selective LBB capture results in delay of right ventricular (RV) activation due to loss of RV depolarization via direct septal myocardial activation. RV activation proceeds via transseptal conduction from left septal fascicles. Electrocardiographic markers of delayed RV activation are V_1_ R-wave peak time prolongation and increase in V_6_-V_1_ interval. (*B*) Transition from non-selective LBB capture to septal does not influence right ventricular activation but delays left ventricular activation due to loss of direct LBB capture.

**Figure 2:**
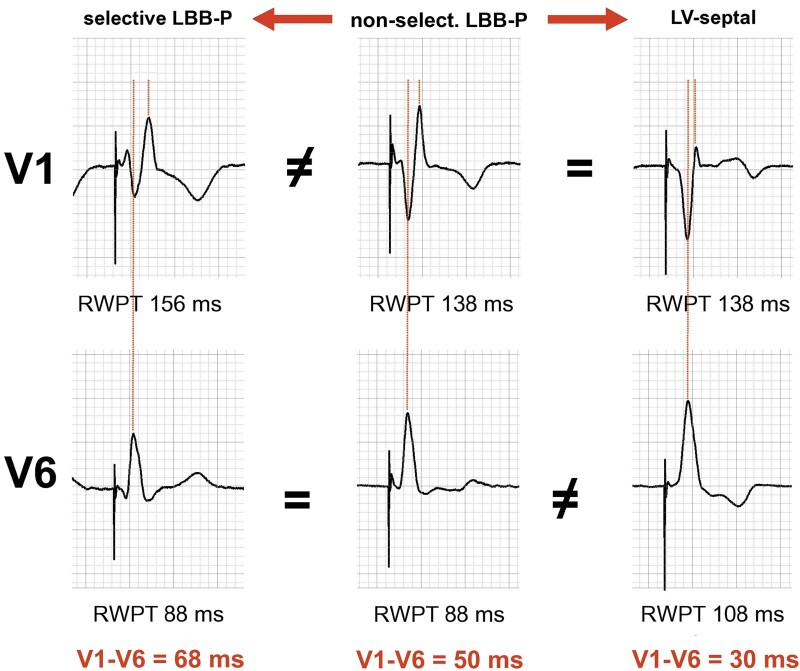
Three types of capture during left bundle branch area pacing (LBB-P) observed in the same patient during pacemaker implantation; leads V_1_ and V_6_ are recorded simultaneously. During the transition from non-selective LBB-P to selective LBB-P, there is an increase in V_1_ R-wave peak time (V_1_RWPT) but not in V_6_RWPT, resulting in a wider V_6_-V_1_ interpeak distance. In contrast, during the transition from non-selective LBB-P to left ventricular septal (LVS) myocardial capture there is an increase in V_6_RWPT but not in V_1_RWPT, resulting in a shorter V_6_-V_1_ interpeak distance.

We intended to develop a new QRS-based criterion that would omit the initial latency that is present after the pacing stimulus and would be measured from an individualized intra-QRS reference time point instead. We hypothesized that the various combinations of direct capture/non-capture of the interventricular septum and capture/non-capture of the LBB result in several distinct patterns of the right ventricular (RV) and left ventricular (LV) activation (*Figure 1*). This could translate into distinct timing of the intrinsicoid deflection in leads V_1_ (a surrogate for RV activation delay) and V_6_ (reflecting LV activation delay). Consequently, the V_6_-V_1_ interpeak interval could differentiate the three types of LBB area capture (*Figure 2*).

## Methods

The study adhered to the Helsinki Declaration, all patients gave written informed consent for participation in this study and the Institutional Bioethical Committee approved the research protocol.

### Aim

Our aim was to analyse the V_6_-V_1_ interpeak interval as a potential new ECG criterion for the differentiation of various types of LBB area capture. In addition, the RWPT in V_1_ and V_6_ were analysed separately during different types of capture to formulate mechanistic hypotheses for the findings.

### Population

Consecutive patients who received LBB area pacing device for bradycardia and/or heart failure indications were screened. The LBB implantation procedure was described by us and others in detail elsewhere.^3–6^ To develop LBB capture criteria, we included only patients with direct evidence of LBB capture obtained during dynamic ECG manoeuvres—this served as a diagnostic ‘gold standard’ in the current study. Such a situation was considered to take place when the paced QRS morphology in lead V_1_ was of the QR/rSR’ type and at least one of the three types of dynamic QRS morphology change below was observed during the procedure (*Figure 1*):

1. Transition from ns-LBB to s-LBB capture during decrease in pacing output.
2. Transition from ns-LBB to LVS capture during decrease in pacing output.
3. Transition from ns-LBB to s-LBB capture during programmed stimulation and/or burst/incremental pacing.^6^
4. Diagnostic pacing was performed only in unipolar pacing mode to avoid the confounding phenomenon of anodal capture.^1^

### QRS measurements

Implantation procedures were recorded on the digital electrophysiological system (LabsystemPRO, Boston Scientific, USA). To ensure high precision, the measurements were performed using all 12 surface ECG leads recorded simultaneously, digital calipers, fast sweep speed (200 mm/s), and appropriate signal augmentation. At least three QRS complexes were measured and the values were averaged.

In each studied patient, every available paced QRS type (s-LBB, ns-LBB and LVS) and native QRS were measured. The following QRS characteristics were obtained:

1. Global QRS duration, measured from the pacing stimulus or QRS onset (in case of intrinsic rhythm or s-LBB capture) to the final QRS component in any of the 12 ECG leads.
2. V_1_RWPT, measured from the pacing stimulus to the peak of the dominant R wave in lead V_1_.
3. V_6_RWPT, measured from the pacing stimulus to the peak of R wave in lead V_6_.
4. V_6_-V_1_ interpeak interval, measured from the R-wave peak in lead V_6_ to the R-wave peak in lead V_1_ during simultaneous recording of all 12 ECG leads (*Figures 1 and* *2*).

Measurements from the QRS onset were used for the native QRS and s-LBB paced QRS.

### Statistical analysis

Continuous variables are presented as means and standard deviations. The distribution of the QRS, V_6_-V_1_ interpeak interval and RWPT in lead V_1_ and V_6_ was estimated by the kernel method. Categorical variables are presented as percentages. Between-group differences were assessed using the Fisher’s exact test for 2 × 2 tables, Student’s *t*-test, or analysis of variance, as appropriate. The performance of binary decision rules was described using sensitivity (SN) and specificity (SP). The performance of the QRS duration and V_6_RWPT in discriminating between ns-HB and RV pacing was assessed using the receiver operating characteristic (ROC) curve. Bland–Altman statistics were then used to assess the inter-observer agreements of QRS duration interval measurements. Statistical analyses were performed using ‘R’ software (The R Foundation). *P*-values <0.05 were considered statistically significant.

## Results

### Population

A total of 468 patients with LBB area pacing from two centres were screened, out of which 124 cases with confirmed diagnosis of LBB capture were included in the further analysis. The remaining cases were excluded as the LBB capture was confirmed only with some non-direct arbitrary criteria (LBB potential, V_6_RWPT, QRS duration, etc.). The basic clinical and procedure-related characteristics of the included patients are presented in *Tables 1 and* *2*. A total of 239 ECG tracings were analysed: 124 with ns-LBB capture, 69 with transition to s-LBB capture, and 46 with transition to LVS capture.

**Table 1:** Basic characteristics of the studied group (*n* = 124)

Age (years)	74.7 ± 11.4
Male gender	71 (57.3%)
Pacing indication (*n*)	
Sick sinus syndrome	32 (25.8%)
Atrioventricular block	41 (33.1%)
Atrial fibrillation with bradycardia	13 (10.5%)
Heart failure	38 (30.6%)
Comorbidities (*n*)	
Diabetes mellitus	49 (39.5%)
Coronary heart disease	51 (41.1%)
Heart failure	59 (47.6%)
Hypertension	99 (79.8%)
Severe valvular disease	16 (12.9%)
Left ventricular ejection fraction (%)	46.2 ± 15.5
Left ventricular end-diastolic dimension (mm)	53.0 ± 8.7
Native QRS duration (ms)	133.6 ± 34.8
Native QRS type	
Narrow	39 (31.6%)
LAFB	5 (4.0%)
RBBB	13 (10.5%)
RBBB + LAFB/LPFB	15 (12.1%)
NIVCD	15 (12.1%)
LBBB	18 (14.5%)
Escape with RBBB-type QRS	13 (10.5%)
Escape with LBBB-type QRS	2 (1.6%)
Complete pacemaker dependency	4 (3.2%)

LBBB, left bundle branch block; LAFB, left anterior fascicular block; LPFB, left posterior fascicular block; NIVCD, non-specific intraventricular conduction disturbance; RBBB, right bundle branch block.

**Table 2:** Pacing- and procedure-related characteristics

	*n* = 124
LBB capture diagnosis based on	
Transition to s-LBBP @TT	69 (55.6%)
Transition to LVS @TT	46 (37.1%)
Selective response @PS	77 (62.1%)
LBB potential observed	71 (57.2%)
Acute ventricular sensing (mV)	8.6 ± 4.7
Acute LBB capture threshold (V)	0.81 ± 0.4
Non-selective LBB QRS (ms)^a^	154.5 ± 21.2
Selective LBB QRS (ms)^b^	144.5 ± 24.4
LV septal QRS (ms)^a^	159.3 ± 20.2
Fluoroscopy time (min)	11.4 ± 9.3
Type of implanted device	
DDD/VVI pacemaker	93 (75.0%)
CRT device	31 (25.0%)

@PS, at programmed stimulation; @TT, at threshold test; CRT, cardiac resynchronization therapy; LBB, left bundle branch; LVS, left ventricular septal myocardial pacing; s-LBBP, selective left bundle branch pacing.

a Measured from the pacing stimulus onset.

b Measured from CQRS onset.

### Categorization of various types of capture during LBB area pacing based on V_6_-V_1_ interpeak interval

**Figure 3:**
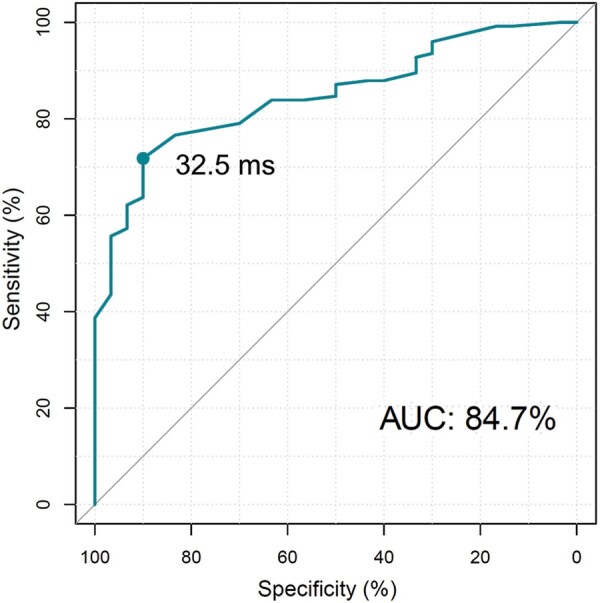
Receiver operating characteristic curve for lead V_6_-V_1_ interpeak interval for the diagnosis of non-selective left bundle branch capture vs. left ventricular septal capture; the V_6_-V_1_ interpeak interval value of 32.5 ms was found as an optimal cut-off point with a sensitivity of 71.8% and a specificity of 90.0%. AUC, area under the curve.

**Figure 4:**
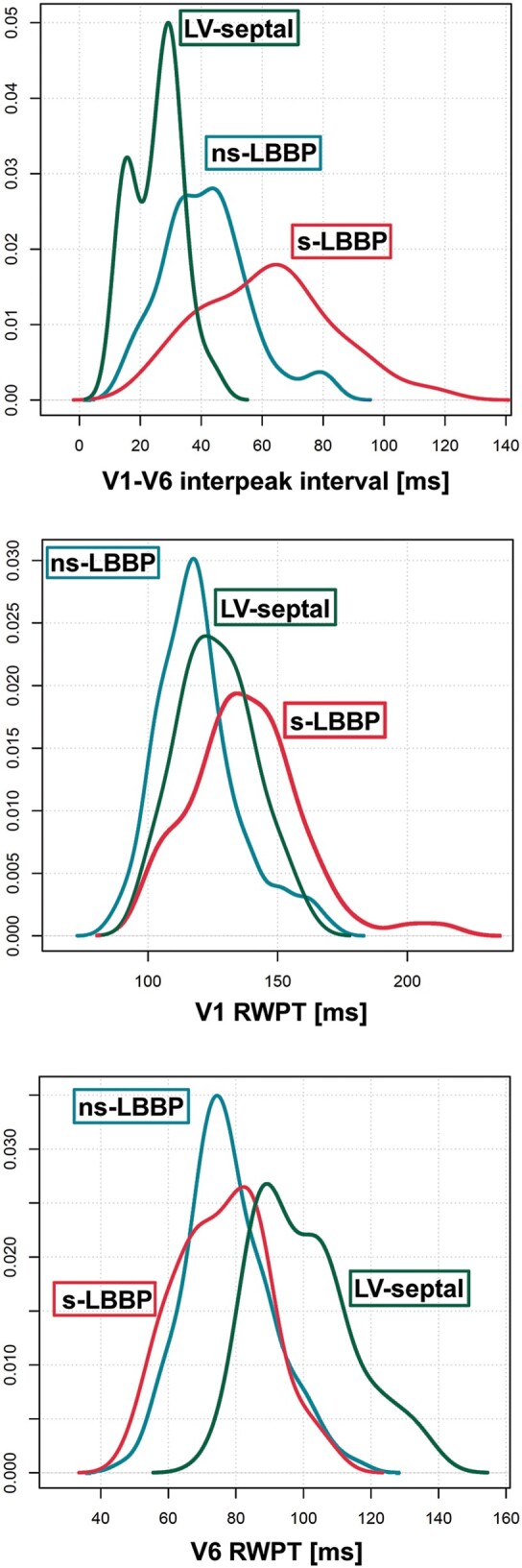
The distribution of V_6_-V_1_ interval values (upper panel) shows better separation between different left bundle branch area capture types than either the distribution of V_1_RWPT (middle panel) or V_6_RWPT (lower panel). Although there is a considerable overlap of V_6_-V_1_ interval values (upper panel), as short interval is observed with all types of left bundle branch area capture, long values (indicating larger RV delay) are only possible with LBB capture and this makes this criterion diagnostically valuable.

The V_6_-V_1_ interpeak interval was longest during s-LBB capture (62.3 ± 21.4 ms), intermediate during ns-LBB capture (41.3 ± 14.0 ms), and shortest during LVS capture (26.5 ± 8.6 ms). The ROC curve for the differential diagnosis of ns-LBB and LV septal capture is presented in *Figure 3*, and the distribution of V_6_-V_1_ interpeak interval values for different LBB area capture types is presented in *Figure 4*. The diagnostically optimal V_6_-V_1_ interpeak interval value for the differentiation of ns-LBB and LVS pacing was 33 ms (SN and SP of 71.8% and 90.0%, respectively). A SP of 100% for the diagnosis of LBB capture was obtained with a cut-off value >44 ms, albeit at the cost of low SN (*Figure 4*).

### V_1_RWPT behaviour during transition from ns-LBB capture to other types of LBB area capture

The distribution of V_1_RWPT values for different LBB area capture types is presented in *Figure 4*.

During transition from ns-LBB to s-LBB capture, the average increase in the V_1_RWPT was 17.8 ± 10.0 ms (120.7 ± 16.7 ms vs. 138.5 ± 21.5 ms, p 0.001); while during transition from ns-LBB to LVS capture, the V_1_RWPT increased by only 6.2 ± 6.3 ms (119.3 ± 14.5 ms vs. 125.6 ± 13.8 ms, *P* < 0.001).

An arbitrary criterion of increase in V_1_RWPT ≥15 ms was fulfilled during transition from ns-LBB to s-LBB and from ns-LBB to LVS capture in 41/69 (59.4%) and 2/46 (4.3%) patients, respectively. Consequently, the SN and SP of this criterion for the diagnosis of transition to selective LBB capture were 59.4% and 95.6%, respectively.

### V_6_RWPT behaviour during transition from ns-LBB capture to other types of LBB area capture

The distribution of V_6_RWPT values for different LBB area capture types is presented in *Figure 4*.

During transition from ns-LBB to s-LBB, the V_6_RWPT remained nearly the same (77.2 ± 13.6 ms vs. 76.6 ± 14.1 ms, p = 0.36); while during transition from ns-LBB to LVS capture, the V_6_RWPT was longer during LVS pacing than during ns-LBB pacing by 19.9 ± 6.7 ms (78.4 ± 10.8 ms vs. 98.4 ± 13.9 ms).

An increase in V_6_RWPT ≥15 ms was observed in 38/46 (82.6%) cases during transition from ns-LBB to LVS capture and in none during transition to s-LBB capture. Consequently, an arbitrary criterion of increase in V_6_RWPT ≥15 ms for the diagnosis of transition to LVS capture had an SN and an SP of 82.6% and 100%, respectively.

### Ancillary analyses

#### Relationship between the V_6_-V_1_ interpeak interval and V_6_RWPT

**Figure 5:**
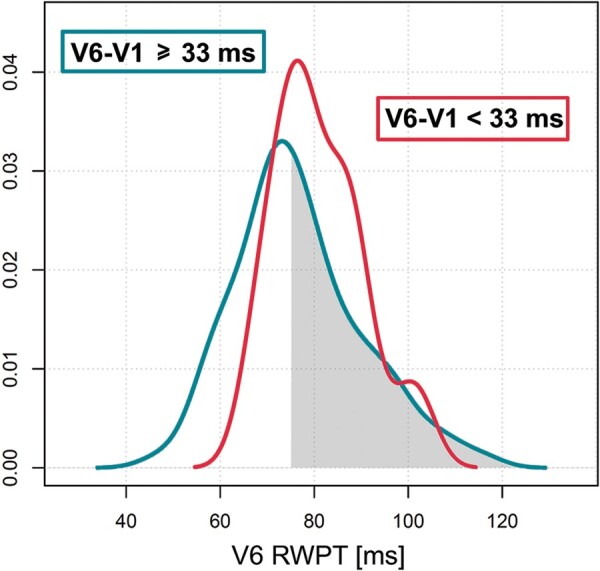
The distribution of V_6_ R-wave peak time (RWPT) values during non-selective left bundle branch capture in patients with V_6_-V_1_ interpeak interval ≥33 ms (blue line) and V_6_-V_1_ interpeak interval <33 ms (red line). In approximately half of the patients with V_6_-V_1_ interpeak interval indicating left bundle branch capture (i.e. ≥33 ms, blue line), the V_6_RWPT was >75 ms (shaded area), indicating left ventricular septal myocardial capture and leading to misdiagnosis.

The V_6_-V_1_ interpeak interval was not related to the paced V_6_RWPT. The distribution of the V_6_RWPT values was similar in patients with diagnostic and non-diagnostic values of the V_6_-V_1_ interpeak interval (see *Figure 5*). Consequently, the V_6_-V_1_ interpeak interval was able to correctly re-classify 44/69 (63.8%) misdiagnosed cases (categorized as false negative by a V_6_RWPT value >75 ms). This V_6_RWPT value was found as indicative of lack of LBB capture with SP of 98.6% and SN of 41%.^7^ Use of both criteria together, i.e. a combined LBB capture criterion (either V_6_RWPT <75 ms or V_6_-V_1_ ≥33 ms) had SP of 94.2% and SN of 78.2%.

#### Analysis of cases where transition from ns- to s-LBB capture did not result in prolongation of V_1_RWPT

In 14/69 (20.3%) patients, transition to s-LBB capture did not result in an increase in V_1_RWPT (difference ≤ 10 ms). In 4 of these 14 patients (28.7%), LBB potential to QRS interval was ≥30 ms. In contrast, only 1/55 (1.8%) patients in whom V_1_RWPT increased by >10 ms had LBB potential to QRS interval ≥30 ms (*P* = 0.005).

#### Impact of native QRS type on V_1_RWPT, V_6_RWPT, and V_6_-V_1_ interpeak interval during non-selective LBB capture

**Figure 6:**
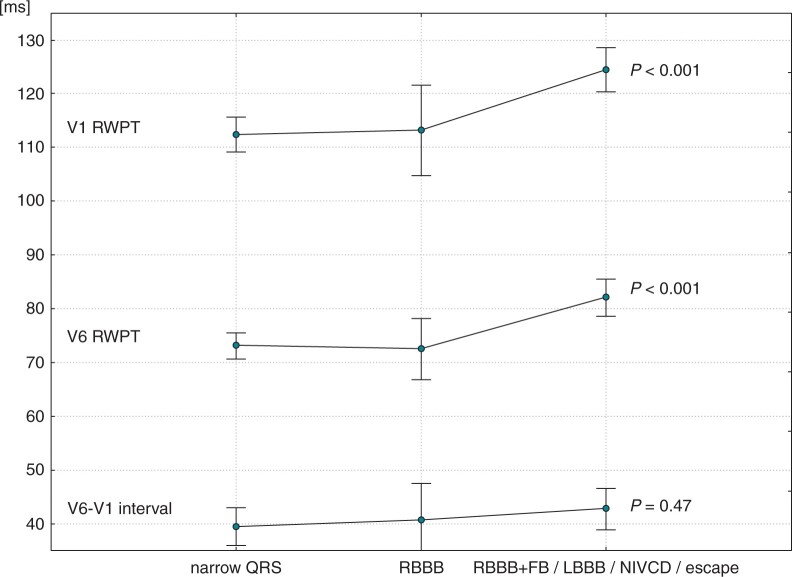
Impact of native QRS type on V_1_ R-wave peak time (V_1_RWPT), V_6_RWPT, and V_6_-V_1_ interpeak interval in patients with narrow QRS, right bundle branch block (RBBB), and more diseased conduction system, including RBBB with anterior or posterior fascicular block (FB), left bundle branch block (LBBB), non-specific intraventricular conduction disturbance (NIVCD), or ventricular escape rhythm/asystole. In patients with more severely diseased conduction system, the V_1_RWPT and V_6_RWPT were prolonged to a similar degree; consequently, their difference, the V_6_-V_1_ interval, remained stable, that is it was not affected by the type of native QRS. Measurements were done during non-selective left bundle branch capture.

The presence of a diseased conduction system (LBBB/NIVCD/RBBB+fascicular block/asystole/ventricular escape rhythm) was related to longer V_6_RWPT and longer V_1_RWPT (*P* < 0.001), while the presence of RBBB did not influence ns-LBB paced V_1_RWPT or V_6_RWPT. Importantly, the V_1_RWPT (measured from QRS onset) during intrinsic rhythm with RBBB was the same as during s-LBB pacing (106 ± 11.7 ms vs. 105.0 ± 14.4 ms, respectively, *P* = 0.60). In contrast to V_1_RWPT and V_6_RWPT, the V_6_-V_1_ interpeak interval was not influenced by the native QRS type (*P* = 0.47). The above relationships are presented in *Figure 6*.

#### Stimulus to QRS latency

Some degree of latency (i.e. an interval between the pacing spike and QRS onset) was observed in all cases. Substantial variability in latency was observed both during ns-LBB and LVS capture and with significant correlation between latency and V_6_RWPT in both capture types (Supplementary material online, *Figures S2* and *S3*). In contrast, V6-V1 interval did not correlated with the latency interval. 

#### Interobserver agreement

Interobserver variation in measurement of V_6_RWPT and V_6_-V_1_ interpeak interval was assessed by comparing the first observer measurements with 224 consecutive measurements by a second independent observer. For V_6_RWPT, the mean difference [95% confidence interval (CI)] was 0.47 ms (−0.24; 1.17) and the mean limits of agreement were –5.55 and 6.48 ms. For V_6_-V_1_ interpeak interval, the mean difference (95% CI) was 0.97 ms (−0.5; 2.44) and the mean limits of agreement were –11.45 and 13.40 ms. The Bland–Altman plots for measurements by the observers are shown in Supplementary material online, *Figure S1*. Both observers agreed on the presence of LBB capture in 88% of cases.

## Discussion

The major finding of this study is that a straightforward electrocardiographic parameter, the V_6_-V_1_ interpeak interval, can differentiate between LBB capture and LVS myocardial capture. A V_6_-V_1_ interval >33 ms showed an SN of 71.8% and an SP of 90% for confirming ns-LBB capture, while the value >44 ms was 100% specific. Importantly, this criterion was often diagnostic in cases where the V_6_RWPT interval provided false negative results.

An additional finding was that an increase in V_1_RWPT and V_6_RWPT of approximately 20 ms each was observed with loss of LVS myocardial capture and loss of LBB capture, respectively (indicating delayed activation of the RV and of the LV, respectively). This sheds light on the physiology of depolarization during LBB area pacing and served to formulate novel criteria for the diagnosis of transition from ns-LBB capture to s-LBB or LVS capture on the basis of simultaneous assessment of sudden increase in V_1_RWPT and V_6_RWPT.

### Physiological background of the V_6_-V_1_ interpeak interval

During transition from ns-LBB to s-LBB capture, the activation of the RV is delayed (resulting in longer V_1_RWPT) because the direct capture of the interventricular septum is lost, while the activation of the lateral wall of the LV remains unchanged. This leads to an increase in the V_6_-V_1_ interpeak interval. In contrast, transition from ns-LBB to LVS capture has no impact on RV activation as, in both situations, RV activation still proceeds from the same pacing site in the interventricular septum. However, LV activation is delayed due to loss of LBB capture, resulting in longer V_6_RWPT. Consequently, during LVS myocardial capture, the V_6_-V_1_ interpeak interval decreases (*Figures 1 and* *2*).

These phenomena are behind the observation that during LVS myocardial capture, despite lack of direct engagement of the His-Purkinje system, the paced QRS is relatively narrow and without pronounced features of either the right or left bundle branch block pattern. This is because there are two oppositely directed activation wavefronts, one in the RV and the other in the LV, resulting in shortened total ventricular activation time and a substantial cancellation effect in the lead V_1_ (lack of pronounced, broad R’). This activation results in a unique QRS pattern in the precordial leads. Since both the RV free wall and LV free wall activations are delayed, the time to intrinsicoid deflection in both V_1_ and V_6_ is delayed. Consequently, the R-wave peaks in V_1_ and V_6_ occur at more or less the same time. Such a unique QRS pattern stands in contrast to the classic RBBB and LBBB patterns, where there is a substantial delay between the R-wave peaks in leads V_1_ and V_6_.

### Clinical translation

The arbitrary V_6_RWPT criterion, popularly used for LBB capture diagnosis, suffers from some limitations. Firstly, apart from our very recent study, there are no validation studies and data regarding its SN and SP for LBB capture diagnosis.^7^ Secondly, while it is reasonable to believe that the currently used V_6_RWPT cut-off points of 75–80 ms are very specific, they are likely not sensitive enough to diagnose LBB capture in many cases. This is because, despite LBB capture, the V_6_RWPT might be prolonged due to latency, slower propagation via a diseased His-Purkinje conduction system, substantial LV dilatation, or a combination of these factors. The V_6_-V_1_ interpeak interval is likely less influenced by these limitations. If there is substantial initial latency or slow conduction through the myocardium, it will affect, to a similar degree, the timing of the activation of the RV and LV. Consequently, the R-wave peak will be delayed in both V_1_ and V_6_, and the V_6_-V_1_ interpeak interval will not be much affected. In other words, the R-wave peak in V_1_ may provide a better reference time point than the pacing stimulus to assess the timing of the R-wave peak in V_6_. Our results show that while V_6_RWPT and V_1_RWPT were strongly influenced by latency and the degree of the disease of the conduction system, as revealed by the type of the native QRS, the V_6_-V_1_ interpeak interval was not (*Figure 6*, Supplementary material online, *Figures S2* and *S3*).

Our study supports this, showing that in patients with V_6_-V_1_ interpeak indicating ns-LBB capture, the V_6_RWPT indicated myocardial capture only in about half the cases. The distribution of V_6_RWPT values in patients with long and short V_6_-V_1_ interpeak intervals was not very different—this suggests that use of a combined criterion (V6RWPT <75 ms or V6-V1 ≥33 ms) might be diagnostically optimal.

### Diagnosis of the type of QRS transition during LBB area pacing

Sudden prolongation of the V_6_RWPT is a recognized marker of transition from ns-LBB to LVS capture.^3^ However, no data support any specific cut-off value, therefore it is not known how much V_6_RWPT should prolong to be diagnostic of loss of LBB capture. Our data suggest that prolongation by even 15 ms is 100% specific; still, in some patients, loss of LBB capture results in even smaller V_6_RWPT prolongation.

Diagnosis of selective capture during His-bundle pacing is straightforward due to the presence of an evident isoelectric interval after the pacing stimulus. However, during LBB pacing, it is often not possible to rely upon the initial isoelectric interval as a diagnostic feature. This is because, firstly, it is often very short and might be obscured by the post-pacing stimulus artefact, and, secondly, an initial latency interval is also present during LVS myocardial pacing. The criterion of sudden V_1_RWPT prolongation ≥15 ms proposed by the current study might be helpful in such situations, as it is very specific for this type of transition during LBB area pacing, especially considering that other QRS morphological features potentially useful for such diagnosis (increase in width of R wave in V_1_, increase in amplitude of S wave in leads I and V_6_, drop in amplitude of R wave in leads I, II, and V_6_) remain to be validated.

### Limitations

A potential confounder in the above hypothesized RV/LV activation patterns and V_6_-V_1_ interval behaviour during LBB area pacing is the retrograde activation of the right bundle branch.^8^ As the majority of paced patients (64.4% in our study) had RBBB, LBBB or complete atrio-ventricular block, i.e. conditions that likely precluded retrograde conduction via the right bundle branch, our finding may not be applicable to patients with preserved conduction. Nevertheless, the following data support the concept that retrograde activation contributes little to the QRS during ns-LBB capture: (i) patients with narrow QRS complex had minimal difference in V_1_RWPT compared to those with RBBB or LBBB (only 3 ms); (ii) in patients with narrow QRS, transition from ns-LBB capture to selective capture also resulted in significant V_1_RWPT prolongation and obvious broadening of the R' in V_1_; (iii) during intrinsic rhythm with RBBB, the V_1_RWPT was the same as during s-LBB capture and shortened with ns-LBB pacing (i.e. with additional septal myocardial activation).

These data suggest that the dominant way of RV activation during ns-LBB pacing is intraseptal depolarization from the LBB area pacing lead as proposed in *Figure 1*. The supero-basal part of the interventricular septum is quite thin, and the pacing helix is partially intraseptal and not completely on the LV side. At the same time, the pathway to the RV via retrograde activation of the conduction system is quite long. Contribution to RV depolarization of retrograde conduction to the right bundle branch probably increases with very proximal LBB lead, close to the His-bundle bifurcation. We have observed that in such cases, characterized by LBB potential to QRS interval of 30–35 ms, transition to s-LBB often results in only minimal V_1_RWPT prolongation.

It is possible that in some cases of LVS capture, there might be nearly instantaneous *secondary* activation of conduction tissue. Such cases might not be differentiated by the V6-V1 criterion from ns-LBB capture, but from a clinical point of view, they may be considered to be equivalent.

The measurements were performed on an EP recording system at 200 mm/s with digital calipers, and other measurement techniques (e.g. using a compass and 25 mm/s printouts) may be less accurate and borderline cases might be difficult to categorize. However, this is a common limitation to all ECG based duration criteria, which are nevertheless commonly used.

## Conclusions

The V_6_-V_1_ interpeak interval was found to be valuable for confirming LBB capture and distinguishing it from pure LVS myocardial capture. The diagnostic performance of a V_6_-V_1_ interpeak delay >33 ms extends beyond the currently used criterion of V_6_RWPT >75–80 ms.

The impact of ns-LBB, s-LBB, and LVS capture on RWPT in V_1_ and V_6_ has provided insight into the physiology of LBB pacing and enabled the development of novel criteria for the differentiation of the three main types of LBB area capture.

## Supplementary material

Supplementary material is available at *Europace* online.

**Conflict of interest:** P.V. has received research and fellowship support as well as speaker and consultant fees from Medtronic. He has also received consultant fees from Abbott, Biotronik, Eaglepoint LLC, and Boston Scientific. M.J. has received consultant fees from Medtronic. K.C. has received consultant fees from Medtronic. P.M. has received consultant fees from Medtronic.

### Data availability

The data that support the findings of this study are available from the corresponding author upon reasonable request.

## Supplementary Material

euab164_Supplementary_Data
