# Supplementary figures and images for: The V6-V1 interpeak interval: a novel criterion for the diagnosis of left bundle branch capture
Source: Europace. 2021 Jul 12;24(1):40–7. doi: 10.1093/europace/euab164 (PMC8742628; doi:10.1093/europace/euab164)

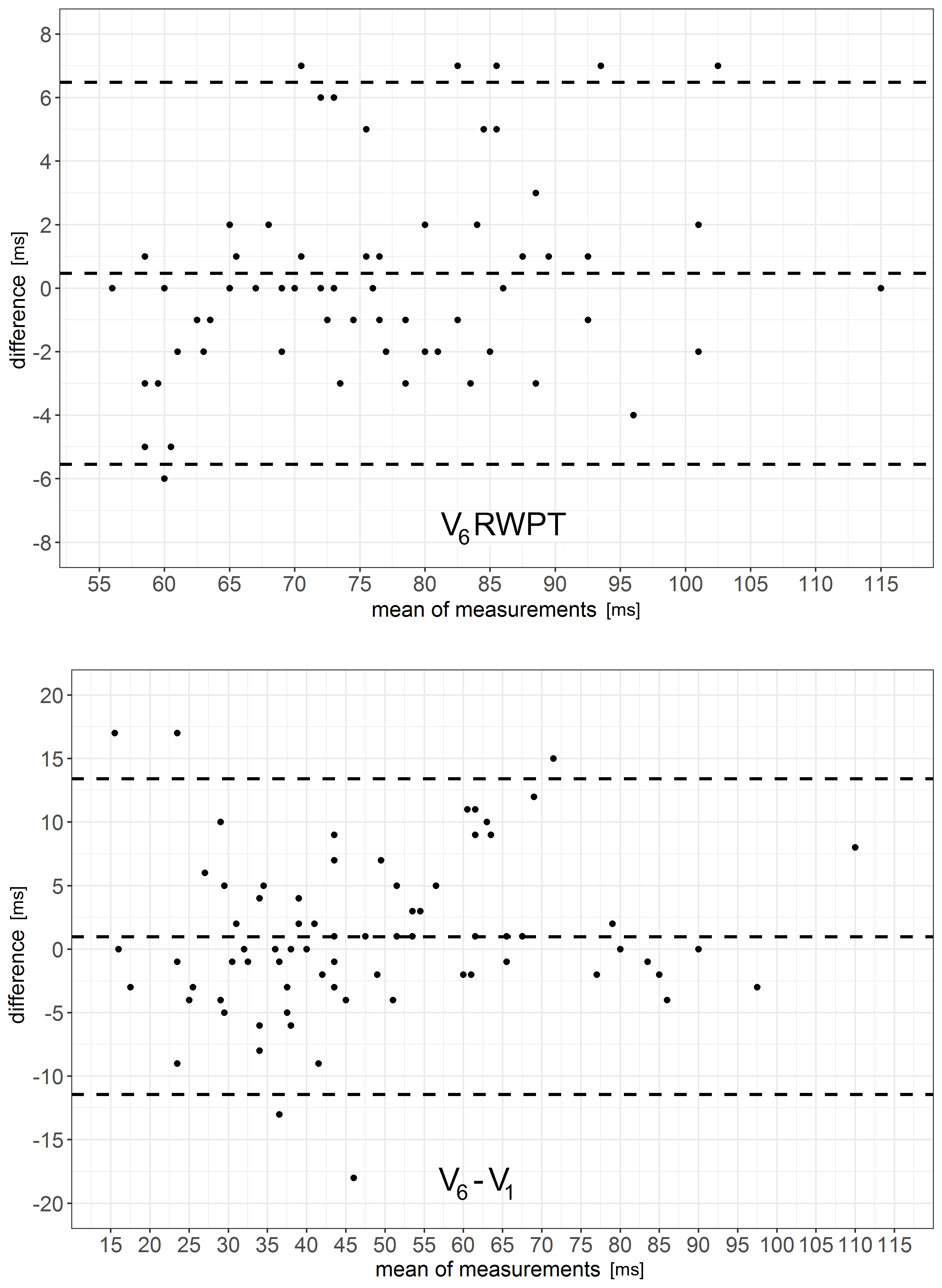

Supplement: euab164_Supplementary_Data [file euab164_supplementary_data.zip › Supplementary Figure 1 R1.jpg]

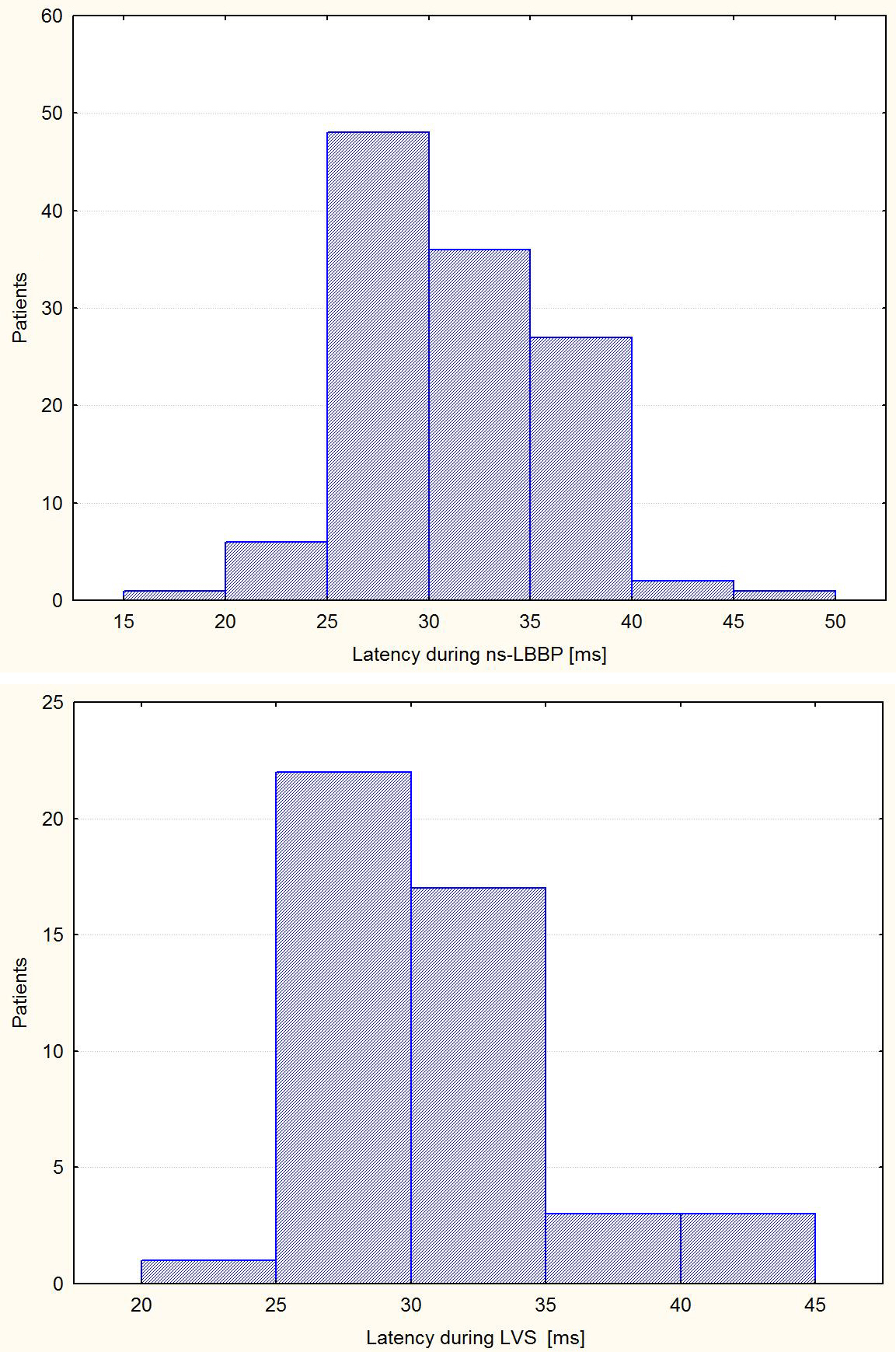

Supplement: euab164_Supplementary_Data [file euab164_supplementary_data.zip › Supplementary Figure 2.jpg]

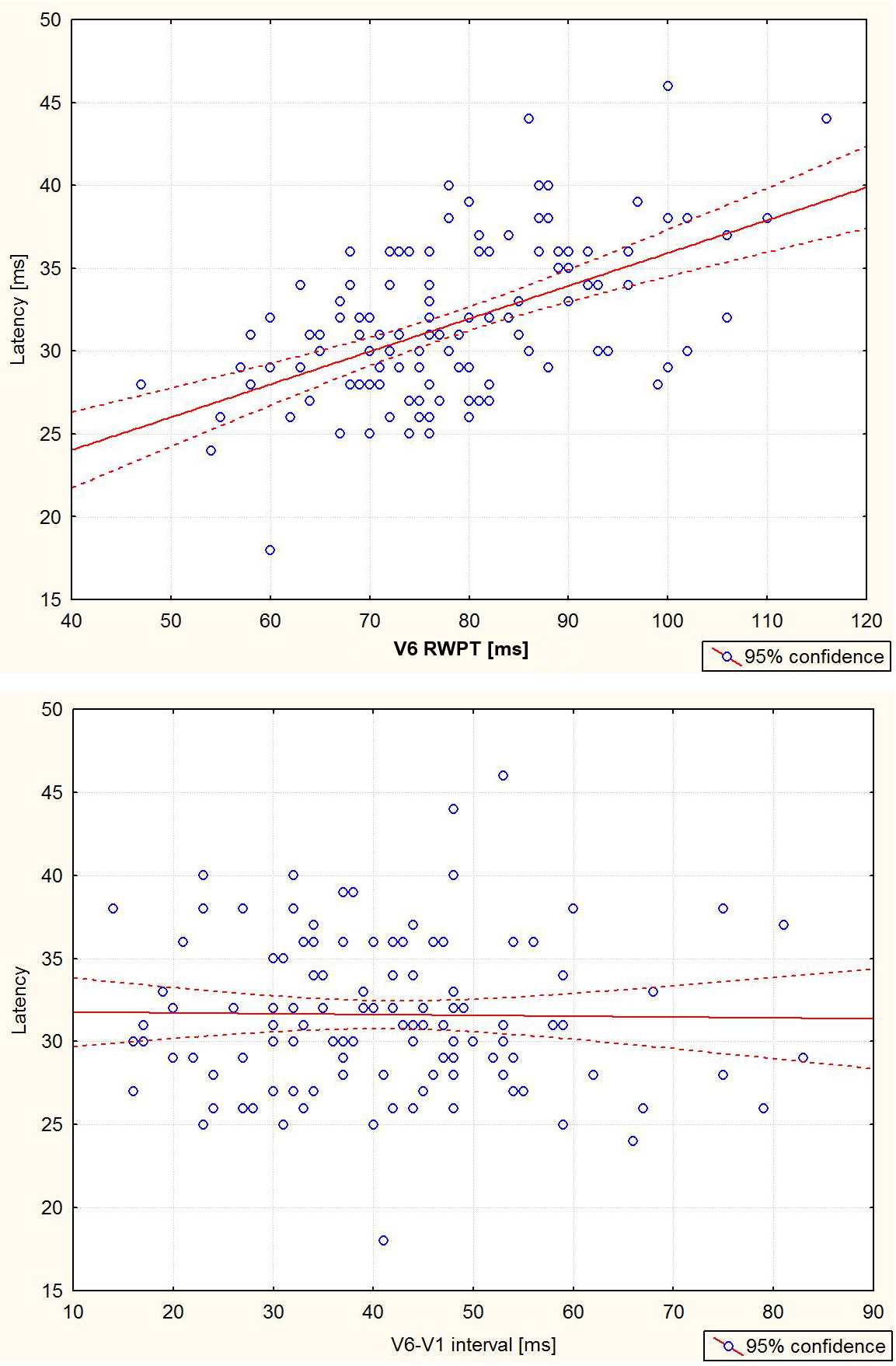

Supplement: euab164_Supplementary_Data [file euab164_supplementary_data.zip › Supplementary Figure 3.jpg]
