# Supplementary material for: The V6-V1 interpeak interval: a novel criterion for the diagnosis of left bundle branch capture
Source: Europace. 2021 Jul 12;24(1):40–7. doi: 10.1093/europace/euab164 (PMC8742628; doi:10.1093/europace/euab164)
Supplement: euab164_Supplementary_Data [file euab164_supplementary_data.zip › Supplementary Figures Legends.doc]

**Supplementary Figure 1.** Bland–Altman plots illustrating difference in R-wave peak time (upper panel) and V_6_-V_1_ interval (lower panel) measurements repeated by a second independent observer in 112 patients. The bias (mean difference) and 95% limits of agreement are shown by the dotted lines.

**Supplementary Figure 2**. Distribution of latency intervals (stimulus to real QRS onset) during non-selective left bundle branch pacing (ns-LBBP) and during left ventricular septal (LVS) myocardial only capture.

**Supplementary Figure 3.** Correlation between R-wave peak in lead V_6_ (V_6_ RWPT) and latency (r = 0.533, p < 0.001) and between V_6_-V_1_ interval and latency (r = - 0.015, p = 0.869) during non-selective left bundle branch capture.
